# Demographic and clinical characteristics of childhood and adult onset Vernal Keratoconjunctivitis in a tertiary care center during Covid pandemic: A prospective study

**DOI:** 10.22336/rjo.2022.61

**Published:** 2022

**Authors:** Arti Singh, Jagriti Rana, Shashwat Kataria, Chandra Bhan, Priyanshi Priya

**Affiliations:** * Department of Ophthalmology, MotiLal Nehru Medical College, Prayagraj, India

**Keywords:** giant papillae, shield ulcer, Horner-Tranta’s spots, conjunctival pigmentation, Keratoconus

## Abstract

**Objective:** To study the demographic and clinical characteristics of childhood and adult onset vernal keratoconjunctivitis in a tertiary care center during Covid pandemic.

**Methods:** A prospective, hospital-based study including a total of 135 patients with Vernal Keratoconjunctivitis (VKC) studied from June 2021 to June 2022 in a tertiary care center in India.

**Results:** Patients presented were in the age range of 1½ to 30 years old. Adult onset VKC included 10.4% of the total patients. The disease was found to be more common in males, with a male to female ratio of 2.5:1. Limbal VKC was the most common presentation found in 61.5% of the patients followed by palpebral type in 28.9% and mixed type in 10.4% of the patients. Severity wise moderate intermittent form of the disease was found to be the most common in 49.6% of the patients. Steroid induced glaucoma was found to be the most common complication in 8.89% of the patients, requiring regular monitoring of intraocular pressure and change in treatment regime, followed by shield ulcer in 2.96% and limbal stem cell deficiency and Keratoconus in 1.48%.

**Conclusion:** This study represents the nature of Vernal Keratoconjunctivitis and its presentation in North India, showing regional variations in the presentation of the disease due to different environmental conditions and the variations in aggression in management protocol. It also emphasizes the regular monitoring of intraocular pressure and the supervision on rising Adult onset VKC.

## Introduction

Vernal keratoconjunctivitis (VKC) is a chronic allergic disease, usually with bilateral presentation and typical ocular symptoms and clinical features such as itching, papillary hyperplasia, gelatinous limbal thickening, Horner Tranta’s spots, though the disease presentations vary in different parts of the world [**1**,**2**,**3**]. The disease is common in the Mediterranean region, South Africa, India and South America [**4**,**5**]. According to most of the studies, it is common in males and more frequently affect the pediatric age group [**6**,**7**], but it may rarely persist beyond puberty or sometimes may begin as adult onset VKC. The severity of the disease also varies in different parts of the world and so does its management. Various grading systems classified the severity of disease, amid the most commonly adopted methods being VKC-CLEK, as described by Bonini et al. and Gokhale et al. [**8**-**10**].

According to most of the reported studies in literature, the demographic and clinical presentations were found to be varied in different regions of the world. The aim of this prospective study was to look for demographic and clinical presentations of patients from this part of the world, where the disease is highly prevalent, but still largely unreported.

## Material and method

A prospective, hospital-based study was conducted at a tertiary care center in Prayagraj, North India, from June 2021 to June 2022, after obtaining clearance from the institutional ethical committee.

A total of 135 patients were identified and diagnosed with Vernal Keratoconjunctivitis. Diagnosis was made based on clinical findings typical of Vernal Keratoconjunctivitis. Patients presented with itching, photophobia, watering, redness in the eyes and on examination had gelatinous limbal thickening, Horner-Tranta’s spots, papillae in palpebral conjunctiva and conjunctival pigmentation. On follow up, patients with inactive papillae, no discharge and complain of itching were diagnosed as having an evolution of the disease. 

Vernal Keratoconjunctivitis was categorized into three types: limbal, palpebral and mixed. Palpebral form of Vernal Keratoconjunctivitis was diagnosed as having papillae of more than or equal to 1 mm with minimal or no limbal infiltrates, limbal Vernal Keratoconjunctivitis as limbal infiltrate with less than 1 mm of palpebral papillae and the mixed form presenting features of both.

Grading was done into mild intermittent, moderate intermittent, moderate persistent, severe persistent and very severe disease by the method described by Bonini et al. [**8**]. Shield ulcer and limbal stem cell deficiency were categorized as very severe.

We collected all details on age of presentation, age of onset, gender, presenting complaint, duration of disease, perennial or seasonal disease, personal and family history of allergy, medications, type and grading of Vernal Keratoconjunctivitis, intraocular pressure, fundus examination, response to treatment and complications related to Vernal Keratoconjunctivitis and its treatment.

The disease was categorized as perennial when it had frequent recurrences throughout the year and seasonal when an acute episode occurred during a particular season.

To define the adult onset of Vernal Keratoconjunctivitis, a fixed cutoff age of 15 years was chosen, with the assumption that at this age, most patients have already passed puberty [**11**]. Those patients who had developed features of Vernal Keratoconjunctivitis in the younger age group that persisted after puberty were excluded from adult onset Vernal Keratoconjunctivitis.

## Results

**Graph 1:**
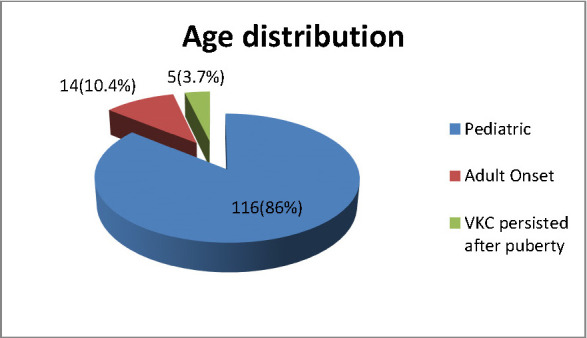
Age distribution of Vernal Keratoconjunctivitis (VKC)

**Fig. 1:**
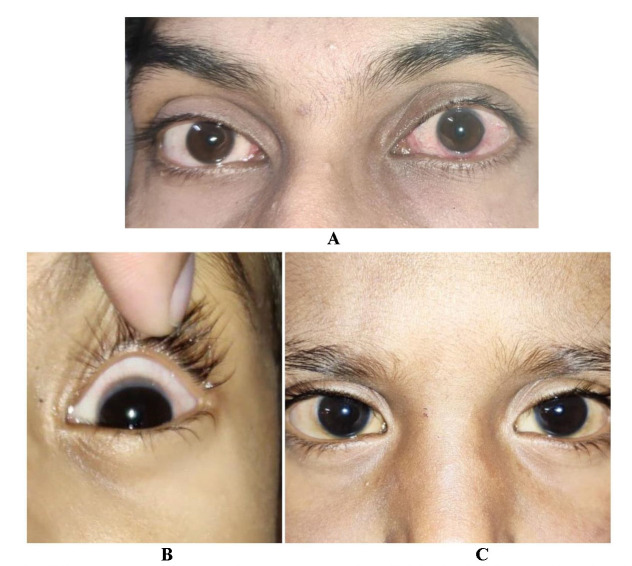
Different presentations of Vernal Keratoconjunctivitis. **A.** A 25-year-old male with adult onset vernal keratoconjunctivitis with gelatinous limbal thickening. **B.** A 4-year-old boy with active limbal vernal keratoconjunctivitis in right eye and quiescent stage in left eye. **C.** A 3-year-old boy with disease evolution in both eyes with perilimbal conjunctival pigmentation

Patients who presented were between 1½ and 30 years old. 71.1% of the patients were younger than 10 years old. The youngest age of onset was 1 year. Adult onset disease was found in 14 patients (10.4%) while disease persisted after puberty in 5 patients (3.7%). The mean age of onset and mean age of presentation were 8.33 ± 6.32 and 10.45 ± 7.19 respectively (**Graph 1**, **Fig. 1**).

96 patients (71.1%) were males as compared to 39 female patients (28.9%). Male to female ratio was 2.5:1.

115 patients (85.2%) had a duration of the disease less than 3 years and in 52.6% of the patients the duration of disease was found to be less than 1 year.

24 patients (17.8%) had a positive family history and 20 patients (14.8%) had a positive personal history with other allergic conditions. Allergic rhinitis was the most commonly associated allergic condition (8.9%), followed by skin allergy (4.5%), and asthma (1.5%) (**Table 1**).

**Table 1:** Demographic characteristics of patients enrolled in the study

	Patients	
Demographic characteristics	Number	Percentage%
Age of onset		
≤ 4 years	44	32.6
5-10 years	52	38.5
11-15 years	25	18.5
≥16 years	14	10.4
Age of presentation		
≤ 4 years	31	23
5-10 years	58	43
11-15 years	27	20
≥16 years	19	14
Gender		
Male	96	71.1
Female	39	28.9
Duration of disease		
≤ 1 Years	71	52.6
1-3 years	44	32.6
3-5 years	10	7.4
≥ 5 years	10	7.4
Family history		
Asthma	6	4.5
Skin allergy	6	4.5
Allergic rhinitis	2	1.5
VKC	10	7.4
Total	24	17.8
Personal history		
Asthma	2	1.5
Skin allergy	6	4.5
Allergic rhinitis	12	8.9
Total	20	14.8

Itch was the most common complaint found in 131 patients (97%), followed by redness in 73 patients (54.7%), watering in 62 patients (45.9%), discharge in 19 patients (14.7%), photophobia in 15 patients (11.1%) and pain in 8 patients (5.92%).

83 patients (61.5%) presented with limbal VKC, which was found to be the most common form of the disease, followed by palpebral type in 39 patients (28.9%) and mixed type in 14 patients (10.4%).

**Fig. 2:**
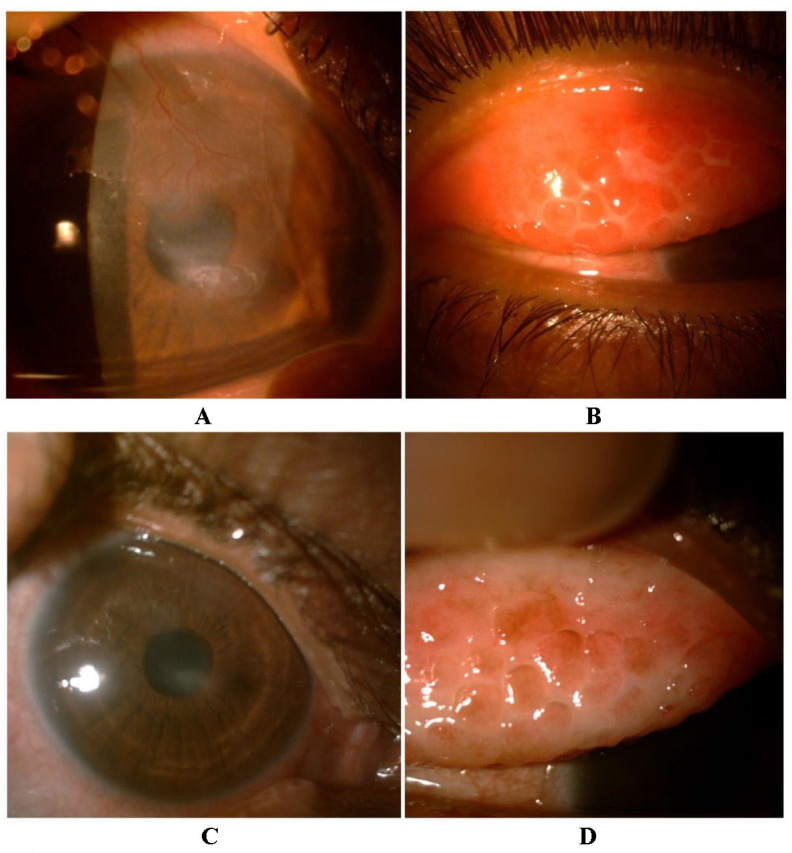
**A.** A 20-year-old male with shield ulcer and limbal stem cell deficiency superiorly; **B.** Cobblestone appearance of upper palpebral conjunctiva; **C.** Healed ulcer with regression of vascularization post supratarsal kenacort injection; **D.** Regression of giant papillae

**Fig. 3:**
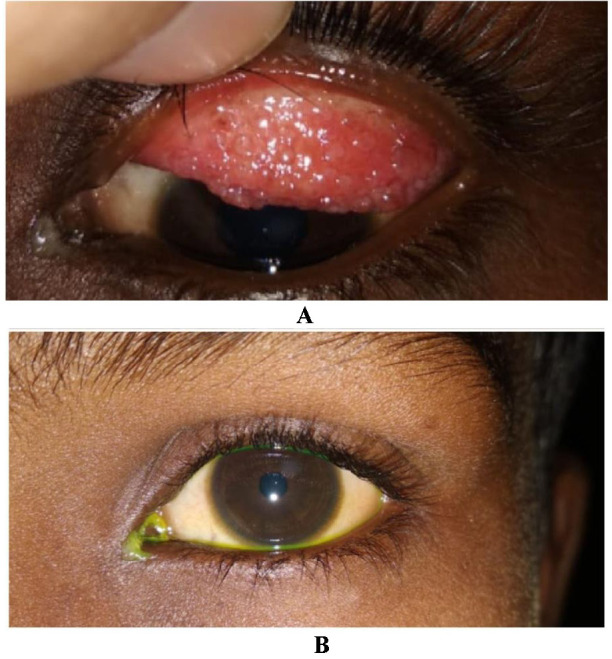
**A.** A 12-year-old child with recurrence of giant papillae after 8 months of papillae excision and amniotic membrane grafting; **B.** Same patient with corneal opacity in superior conjunctiva representing healed shield ulcer

The grading of the severity of the disease was done based on Bonini et al. classification. 67 patients (49.6%) presented with moderate intermittent form of the disease, which was found to be the most common form, followed by mild intermittent in 35 patients (25.9%), moderate persistent in 23 patients (17.3%), severe persistent in 8 patients (5.92%) and very severe in 2 patients (1.5%) (**Fig. 2**,**3**).

85 patients (62.9%) had a visual acuity of 6/ 18 or better. 4 patients (2.96%) had a visual acuity of less than 3/ 60. Conjunctival pigmentation was found in 39 patients (28.9%), while it was absent in 96 patients (71.1%) (**Table 2**).

**Table 2:** Clinical characteristics of patients enrolled in the study

	Patients	
Clinical characteristics	Number	Percentage%
Symptoms		
Itching	131	97
Watering	62	45.9
Redness	73	54.07
Photophobia	15	11.1
Discharge	19	14.07
Pain	8	5.92
Visual acuity		
≥ 6/ 18	85	62.9
< 6/ 18-6/ 60	6	4.45
< 6/ 60-3/ 60	0	0
≤ 3/ 60	4	2.96
Could not be assessed	40	29.6
Type		
Limbal	83	61.5
Palpebral	39	28.9
Mixed	14	10.4
Severity of VKC		
Mild intermittent	35	25.9
Moderate intermittent	67	49.6
Moderate persistent	23	17.03
Severe persistent	8	5.92
Very severe	2	1.5
Conjunctival pigmentation		
Yes	39	28.9
No	96	71.1
Complications		
Steroid induced glaucoma	12	8.89
Cataract	0	0
Corneal scar	4	2.96
Keratoconus	2	1.48
Shield ulcer	2	1.48
Limbal stem cell deficiency	2	1.48

Steroid induced glaucoma was found to be the most common complication in 12 patients (8.89%), who, on treatment, got relieved. We did not find cataract in any of our patients. Corneal scar, which was healed shield ulcer, diagnosed based on its typical location and shape, was found in 4 patients (2.96%), while shield ulcer, limbal stem cell deficiency and keratoconus were found in 2 patients (1.48%) (**Table 3**).

**Table 3:** Posttreatment status of patients

	Patients	
Posttreatment Status	Number	Percentage%
Improved (steroid could be tapered/ stopped)	81	60
On longer steroid treatment ± Tacrolimus	42	31.1
Steroid induced glaucoma	12	8.89

## Discussion

In our study, 96 patients (71.1%) were younger than 10 years old at the onset and 44 patients (32.6%) were younger than 4 years old. We found that although the disease was common in school-aged children, the younger age group was equally affected. Similar to our study, a study conducted by Leonardi et al. in Italy found that 83% of the patients were younger than 10 years old [**12**].

The mean age of presentation in our study was 10.45 ± 7.19 years and the mean age of onset was 8.33 ± 6.32 years. In ten patients who presented with severe and very severe disease, the age of onset was between 5 to 22 years. We did not find any association of severity with age. Similar to our study, another study conducted by Lambiase et al. in South Italy found the mean age of presentation 13.8 ± 8.8 years, while the mean age of onset was 7.47 ± 6.9 years [**13**]. Another study that was performed in Italy by Bonini et al. found that the mean age of presentation was 11 ± 5.8 years, while the mean age of onset was 7.1 ± 4.7 years [**14**]. In a study by S. Saboo et al. in Southern India, during a 1-year period, the mean age of presentation was 12 ± 6.63 years [**15**]. No regional variation was found in the distribution of the disease according to age.

The minimum age of onset in our study was 1 year, and 22 years was the maximum. Lambiase et al. found the minimum age of onset to be 3 years and the maximum 38 years.

Even if VKC is believed to be a disease of childhood, which usually resolves at puberty, we found 19 patients (14%) with the disease above 15 years old. Of these, 14 patients (10.4%) had an adult onset disease, while in 5 patients (3.7%), the disease persisted after puberty. Leonardi et al. found 4% of the patients above 20 years old, while Shafiq et al. found 6% of the patients with VKC above 20 years old [**16**]. Saboo et al. found that 12% of the patients were above 20 years old. A smaller percentage of patients in these studies may be due to their cutoff age of 20 years, while we considered 15 years as the cutoff age.

In our study, 71.1% of the patients were males, showing a male preponderance with male to female ratio of 2.5:1, thus suggesting a hormonal influence in the development of VKC, with an adult onset disease showing slightly more different, with a male preponderance of 4:1. In other studies, male to female ratio was reported between 4:1 to 2:1 (Neumann et al. 1959 [**17**], Tuft et al. 1989 [**18**], Bonini et al. 2000 [**14**], Saboo et al. 2006 [**15**]), showing a global pattern of male preponderance of VKC. Bonini et al. observed a male to female ratio of 3.2:1 of less than 20 years, while it was 4:1 above 20 years old [**14**].

17.8% of our patients had a positive family history and 14.8% had a positive personal history with other allergic conditions. Allergic rhinitis was the most commonly associated allergic condition in 8.9% of the patients. Studies in India do not show a high preponderance of personal and family history, which ranges from 1.6% to 6% (Saboo et al. [**15**], Vdas et al. [**19**], Singh et al. [**20**]) as compared to literature from temperate zones where it ranges from 41.5% to 48.7% (Leonardi et al. [**12**], Lambiase et al. [**13**], Bonini et al.[**14**]) with allergic rhinitis as the most common finding in personal history.

Environmental factors may play a role in these regional differences associated with the same disease, as the patients in tropical countries have shown less associations with personal and family history and usually presented with a perennial form of the disease.

Itching was the most common complaint in our study, 97% of the patients presenting with it. It is similar to other Indian studies in literature. A study by Saboo et al. in Southern India showed 88% of the patients with itching, while one of the studies in the temperate zones, by Lambiase et al. in Italy, showed itching/ burning in 76.8% of the patients.

61.5% of our patients presented with a limbal form of VKC, while 28.9% with palpebral and 10.4% with mixed form. According to Saboo et al. study performed in Southern India, regional differences have been found, showing a mixed form as the most common presentation (71.8%), followed by palpebral (15.6%) and limbal (12.6%). Another study conducted in Northern India by Sofi et al., showing the limbal form as the most common presentation (77%), was similar to our study [**21**]. Data from temperate zones also showed a variable presentation: Leonardi et al. showed that 68.5% of the patients had a palpebral form of disease, Bonini et al. showed that 83.6% of the patients had a palpebral form of disease, while a study by Lambiase et al. showed the limbal form of the disease (53.2%) as the most common presentation [**12**,**13**].

The grading of the severity of the disease is done based on Bonini et al. classification, 49.6% of the patients presenting with moderate intermittent form of the disease as the most common form, followed by mild intermittent (25.9%) and moderate persistent (17%). Patients with adult onset of the disease were not much different from this data, 75% of the patients showing mild to moderate form of disease. Regional differences were found with Saboo et al., showing 32% of the patients with severe and very severe disease, while in our study only 7.42% of the patients presented with this severity.

In our study, we have found 62.9% of the patients with visual acuity of 6/ 18 or better, while 2.96% of the patients had a visual acuity of < 3/ 60. Poor visual acuity in these patients was due to corneal scarring, keratoconus and shield ulcer with LSCD.

We have found two patients (1.48%) with shield ulcer and both were associated with palpebral VKC with giant papillae. The first patient was managed with ulcer debridement, and bandage contact lens with supratarsal Triamcinolone injection and healed in 3 weeks. Another patient with recurrent shield ulcer was managed with papillae excision, with amniotic membrane transplantation and showed recurrence of papillae after 3 months. Saboo et al. in India showed 3% of the patients with shield ulcer, while Das et al. 0.3% [**15**,**19**]. Literature from temperate countries showed an incidence of shield ulcer ranging from 7.7% to 15.3% (Leonardi et al., Lambiase et al. and Bonini et al.) [**12**-**14**]. Even though they found most of their patients with shield ulcer (66.7% to 68.5%) to be associated with a tarsal form of the disease, 11.1% to 16.7% of the patients were associated with a limbal form of the disease.

Limbal stem cell deficiency was present in two patients (1.48%). Saboo et al. found 1.2% of the patients with center involving LSCD. In our patients, conjunctivalization did not involve the center of the cornea and so patients were managed medically.

We found keratoconus in 2 patients (1.48%). However, we did not perform corneal topography regularly in every VKC patient. Das et al. found keratoconus in 1.36% of the patients, while Leonardi observed it in three patients. Topography was not performed on a regular basis in any of these studies.

Out of these 135 patients, 81 patients (60%) showed a significant improvement with treatment, which was given in the form of antihistamines with or without topical steroids. In these patients, steroids could be tapered. 42 patients (31%) were on steroids with or without Tacrolimus .03% ointment and required longer steroid treatment. 12 patients (8.89%) developed steroid induced glaucoma.

Topical Tacrolimus .03% as immunomodulator and steroid sparing drug were used in 53 patients (39.3%). Out of these 31 patients (58.5%) who had a perennial disease, 8 patients (15%) had steroid induced glaucoma and 14 patients (26.4%) were those in whom the tapering of steroids led to the worsening of symptoms. 3 patients (5.7%) stopped ointment due to burning and it was changed to Cyclosporine.

Steroid induced glaucoma was found in 12 patients (8.89%). Out of these, three patients received supratarsal triamcinolone injection, six patients were on eye drop prednisolone, and three patients were on eye drop loteprednol. Six of these patients (50%) had glaucomatous optic disc changes. In all these patients, steroids were slowly withdrawn and anti-glaucoma medication added. None of the patients needed any surgical intervention for raised intraocular pressure. Recurrence of symptoms occurred in all the patients even though Tacrolimus was added as steroid sparing drug.

Saboo et al. showed 18 patients (4%) with steroid induced glaucoma. Out of these, 6 patients required trabeculectomy, and one patient trabeculotomy with trabeculectomy. None of our patients had intractable glaucoma. Bonini et al. found 2% of the patients with steroid induced glaucoma [**14**].

In our study, we did not find cataract in any of our patients. Saboo et al. found cataract in 6.19% of their patients, while Leonardi et al. observed cataract in one of their patients. We found corneal scar in four (2.96%) of our patients.

Conjunctival pigmentation was present in 28.6% of the patients. According to Rao et al., it was both a specific and sensitive sign of VKC. It was also present in quiescent disease, so it helped in the diagnosis of VKC patients.

The strength of our study is its prospective nature as most of the studies in literature were found to be of retrospective nature, whereas the lacuna of our study was its small sample size, this being due to the low footfall in the Covid era.

The patients and the parents need to be counselled about the nature of the disease, long-term requirement of medication and on the physician’s side. Measuring the intraocular pressure is always needed, so that steroid induced glaucoma could be diagnosed and managed as early as possible.

## Conclusion

This study represents the nature of Vernal Keratoconjunctivitis and its presentation in North India, also showing the rising incidence of adult onset. It shows regional variation in presentation and severity of disease due to different environmental conditions and so the aggression in management protocol also varies. 

Further prospective studies with larger sample size and longer follow up are needed to know the nature of the disease and its long-term response to treatment. Moreover, intraocular pressure measurement should be emphasized in every patient on steroids, so that childhood blindness due to steroid induced glaucoma could be avoided.

**Conflict of Interest statement**

The authors state no conflict of interest.

**Informed Consent and Human and Animal Rights statement**

Informed consent has been obtained from all individuals included in this study.

**Authorization for the use of human subjects**

Ethical approval: The research related to human use complies with all the relevant national regulations, institutional policies, is in accordance with the tenets of the Helsinki Declaration, and has been approved by the review board of MotiLal Nehru Medical College, Prayagraj, India.

**Acknowledgements**

None.

**Sources of Funding**

None.

**Disclosures**

None.

**Registration number in case of a clinical trial**

Not applicable.
